# Effects of Grazing and Leaf Spot Disease on the Structure and Diversity of Phyllosphere Microbiome Communities in *Leymus chinensis*

**DOI:** 10.3390/plants13152128

**Published:** 2024-08-01

**Authors:** Yani Qian, Yuanyuan Jin, Xinyao Han, Kamran Malik, Chunjie Li, Binhua Yu

**Affiliations:** 1 Grassland Research Center of National Forestry and Grassland Administration, Chinese Academy of Forestry, Beijing 100091, China; qianyani2024@outlook.com (Y.Q.); 18809499001@163.com (X.H.); 2 State Key Laboratory of Herbage Improvement and Grassland Agro-Ecosystems, Lanzhou University, Lanzhou 730020, China; jinyy20@lzu.edu.cn (Y.J.); malik@lzu.edu.cn (K.M.); 3 College of Pastoral Agriculture Science and Technology, Lanzhou University, Lanzhou 730020, China; 4 Engineering Research Center of Grassland Industry, Ministry of Education, Lanzhou University, Lanzhou 730020, China; 5 Key Laboratory of Grassland Livestock Industry Innovation, Ministry of Agriculture and Rural Affairs, Lanzhou University, Lanzhou 730020, China; 6 Gansu Tech Innovation Center of Western China Grassland Industry, Lanzhou University, Lanzhou 730020, China

**Keywords:** cattle grazing, plant community, fungi disease, phyllosphere microbiome, interaction

## Abstract

*Leymus chinensis* is a high-quality forage with wide distribution. Disease is an important factor affecting the yield and quality of *L. chinensis*. To investigate the effect of grazing on the phyllosphere microbiome community and leaf spot disease in *L. chinensis*, high-throughput sequencing technology was used to study the differences in the composition and structure of the phyllosphere fungal and bacterial communities of healthy and diseased leaves under different grazing intensities. The results showed that grazing significantly reduced leaf spot disease incidence and severity. There were significant differences in the phyllosphere microbiome composition between healthy and diseased leaves, and interestingly, diseased leaves showed more complex microbial activity. Grazing altered the relative abundance of micro-organisms and affected microbial dispersal and colonization either directly through behavior or indirectly by altering plant community structure. In this study, we found that the phyllosphere microbiome responded strongly to pathogen infection, and that plants recruited beneficial microbes to protect themselves after disease development. Grazing could regulate microbial community composition and structure, either directly or indirectly, and plays a crucial role in maintaining the health of *L. chinensis*.

## 1. Introduction

*Leymus chinensis* is a perennial rhizomatous grass widely distributed in northern China, Mongolia, the Korean peninsula, and Siberia (GBIF), with high leaf volume, rich in nutrients, and good palatability, and it is the main high-quality forage grass in northeastern China [1,2,3]. However, it is susceptible to infection by pathogens, which affects its economic and ecological values [1,4]. Grassland plant diseases, such as leaf spot, are the most diverse and widely distributed disease groups. *Leptosphaeria avenaria* and *Parastagonospora nodorum* could lead to the leaf spot disease of *L. chinensis* [1,5]. Thus, the plant biomass, nutrients, as well as growth rate decrease obviously, affecting its yield and quality [4]. Pathogens parasitizing perennial plants also have long-term cumulative impacts on grassland communities, even endangering other plants around the host and changing the direction of vegetation succession in the whole community [6,7,8].

Grazing is the main utilization method of natural grasslands; animal feeding, trampling, and feces may either reduce the incidence of disease or increase the chances of disease transmission and infestation. Animal feeding has the ability to eliminate pathogens by removing pathogen-infected plant tissues, reducing the primary source of infection, and mitigating disease incidence. Additionally, grazing increases the infection of certain pathogens and exacerbates the spread of disease by creating new wounds on plant surfaces [9,10,11]. The movement of herbivores and mechanical damage caused by trampling can also accelerate the spread of some pathogens across grasslands [11]. In addition, animal feces can increase soil nitrogen and phosphorus concentrations, significantly alter soil chemistry, and increase the risk of plants infecting themselves with fungal pathogens [12]. Damage caused by grazing can also cause plant tissues to activate jasmonic acid-dependent defense responses more quickly and efficiently, therefore reducing pathogen invasion [13,14]. At the same time, grazing can also indirectly affect the occurrence of plant diseases by altering the plant community. Studies have shown that due to the selective feeding on plants by herbivores can lead to changes in species composition, canopy characteristics, and the phylogenetic structure of plant communities [15,16]. Microenvironmental factors, such as humidity, temperature, and wind speed, closely linked to disease incidence and pathogen spread are also influenced by grazing [17]. Thus far, many studies have proved that moderate grazing effectively controls certain diseases in natural grasslands by reducing the occurrence of diseases, such as leaf spot diseases, through direct or indirect factors [11,18].

It is well known that microbial communities are closely related to plant health, and beneficial micro-organisms can protect plants from pathogens by antagonizing, competing with pathogens, or establishing a reciprocal, symbiotic relationship with the host plant [19,20]. Whereas, some micro-organisms are potential phytopathogens, and growth and reproduction under suitable environmental conditions can jeopardize plant health [21,22]. Several studies have focused on the relationship among soil, inter-root microbial communities, and plant health, and reported that plant diseases strongly influence microbial community structure, and in turn, microbes influence disease development through complex interactions as well. Jiao et al. (2022) discussed that the diversity of microbial communities in soil samples of healthy *Fritillaria ussuriensis* was higher than that in soil samples of *F. ussuriensis* infected with *Fusarium* sp. [23]. The probable reason could be that the root secretions of healthy plants can provide more nutrients to soil micro-organisms, thus increasing the species richness and diversity of the microbial community. Fernandez-Gonzalez et al. (2020) reported that susceptibility/tolerance to soil-borne pathogens in olive was correlated with changes in the inter-root microbial network [24]. Like soil micro-organisms, phyllospheric micro-organism have similar effects on plant health. The plant leaf provides a vast living space and nutrients for micro-organisms, which can inhibit the growth of plant pathogens through mechanisms, such as nutrient competition and antagonism [25,26,27]. It can also affect host plants through the production of volatile organic compounds (VOCs), produced by the *Poaceae* endophytic fungi, *Epichloë* sp. It has fungitoxic effects at ecologically significant concentrations, where Chokol K inhibits spore germination of plant pathogenic fungi [28]. Thus, plants can maintain health by regulating the phyllospheric micro-organism composition and diversity, as well as preventing flora dysbiosis. Relevant studies have mainly focused on crops and model plants, with greenhouse pot experiments, and little has been reported in complex natural grassland ecosystems [29,30,31,32,33].

Few other studies suggest that animal trampling, selective feeding, and defecation alter the abundance, composition, and diversity of soil micro-organisms by affecting soil nutrient cycling and soil structure, which are dependent on the intensity and duration of grazing [34,35]. Jing et al. (2023) found that yak grazing altered the structure of soil microbial communities [36], however, the network complexity of bacteria and fungi responded differently to grazing. Eldridge and Delgado-Baquerizo (2018) showed that sheep grazing had direct and indirect negative effects on the abundance of plant pathogens [37]. Existing studies of grazing-affected microbial community changes and grazing-affected plant diseases have been conducted independently, with few studies linking the two parts. Consequently, there is a paucity of research on the effects of grazing on microbial communities and plant health, focused mainly on soil micro-organisms. However, the destruction of plant above-ground tissues by feeding and trampling by grazing livestock creates favorable conditions for the colonization of phyllospheric micro-organism. Simultaneously, it may also disrupt the interactions between micro-organisms that have already been effectively colonized, compromising the normal growth and development of the plant. Excreta from domestic animals could also inevitably affect plant phyllospheric micro-organisms through direct contact with plant above-ground tissues or by altering the physiological and biochemical properties of the soil, thereby affecting the normal growth of the plant.

Theoretically, the pathogens that cause leaf spot disease in *L. chinensis* mainly infect the wounds on plant leaves caused by livestock feeding and trampling. Livestock causes wounds more conducive to the horizontal transmission of micro-organisms, and livestock feeding may reduce the load of pathogens and the source of primary infestation in the field. The saliva and excreta of livestock may change the phyllospheric micro-organism community, and some of them may also act as antagonists to the pathogen, parasitism, and other complex relationships. In summary, the microecological balance composed of grazing livestock, pasture, and phyllospheric micro-organism is important for the healthy growth of *L. chinensis* in natural grasslands.

Exploring the interactions and synergistic development of livestock-pasture diseases and phyllospheric micro-organism under grazing conditions could be a meaningful study. Therefore, this study investigated the effects of grazing intensity on the phyllospheric micro-organism community and took a typical leaf spot disease in *L. chinensis*, as the research object. The study was aimed at elucidating the interactions between livestock-quality pasture leaf disease and phyllospheric micro-organism in natural grassland ecosystems. The current research could provide a theoretical basis for the sustainable utilization of natural grassland and the biological prevention and control of high-quality pasture diseases.

## 2. Results

### 2.1. Effects of Grazing on Leaf Spot Disease and Vegetation of Leymus chinensis

**Figure 1:**
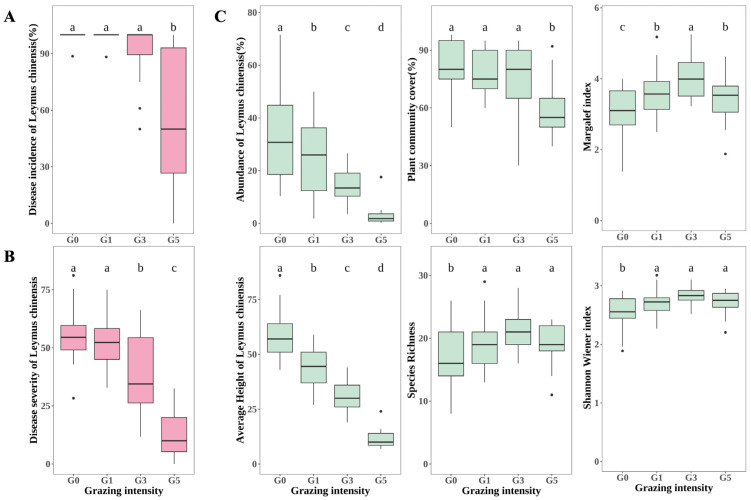
Effects of grazing on plant diseases and ecological indexes of plant communities. (**A**) changes in disease incidence in *Leymus chinensis* leaf spot; (**B**) changes in disease severity in *L. chinensis* leaf spot; (**C**) changes in ecological indexes: abundance in *L. chinensis*, average height of *L. chinensis*, coverage, species richness, Margalef index, and Shannon Wiener index. Different lowercase letters indicate significant differences at *p* < 0.05; G0, 0 standard bovine units per hectare; G1, 0.23 standard bovine units per hectare; G3, 0.46 standard bovine units per hectare; and G5, 0.92 standard bovine units per hectare.

Grazing significantly reduced the incidence as well as the severity of leaf spot disease in *Leymus chinensis* (*p* < 0.05) (Figure 1A,B). The incidence of leaf spot disease of *L. chinensis* was more than 99% in both G0 and G1, and 53.59% ± 4.88 in G5, which was significantly (*p* < 0.05) lower than that of G0, G1, and G3. There were no significant differences in the incidence of *L. chinensis* leaf spot disease between G0, G1, and G3 (*p* > 0.05). The severity of leaf spot disease in *L. chinensis* decreased gradually with increasing grazing intensity, with no significant difference between G0 and G1 (*p* > 0.05), and both G3 (37.45 ± 2.71) and G5 (13.12 ± 3.75) were significantly lower than G0 and G1 (*p* < 0.05).

*L. chinensis* abundance and average height decreased significantly with grazing intensity (*p* < 0.05), and under heavy grazing conditions, *L. chinensis* abundance was almost zero. (Figure 1C). The cover of the entire plant community also decreased gradually with increasing grazing intensity, but there was no significant difference between G0, G1, and G3 (*p* > 0.05), and the cover of the plant community in G5 (62.91% ± 4.30) was significantly lower than that in G0, G1, and G3 (*p* < 0.05) (Figure 1C). As grazing intensity increased, species richness showed a trend of increasing and then decreasing, with G3 (21.14 ± 0.80) having the highest species richness and G0 (16.71 ± 0.80) the lowest, but there was no significant difference in species richness between G1, G3, and G5 (*p* > 0.05). The trends of the Margalef index and Shannon Wiener index were consistent with those of species richness, but the Margalef index was significantly higher in G3 (4.06 ± 0.14) than in G1 and G3 (*p* < 0.05) (Figure 1C).

### 2.2. Microbial Composition

#### 2.2.1. Differences in Microbial Genus Levels across Treatments

**Figure 2:**
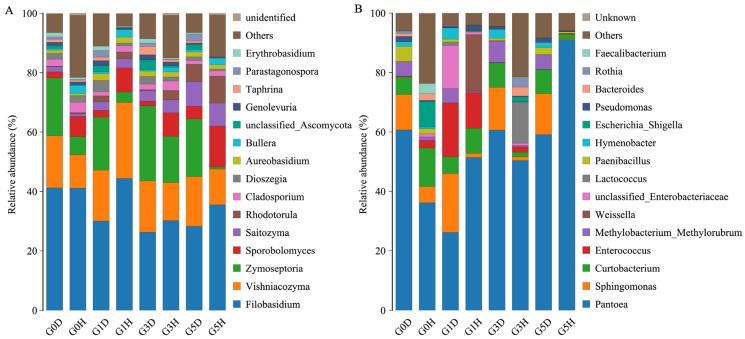
Stacked histograms of genus-level composition of epiphytic fungi (**A**) and epiphytic bacteria (**B**) in *L. chinensis* leaf spot diseased leaves versus healthy leaves under different grazing intensities. D stands for leaf spot diseased leaves, and H stands for healthy leaves. G0, 0 standard bovine units per hectare; G1, 0.23 standard bovine units per hectare; G3, 0.46 standard bovine units per hectare; and G5, 0.92 standard bovine units per hectare.

A total of 8 phylums and 181 genera of epiphytic fungi and 22 phylums and 272 genera of epiphytic bacteria were detected in 24 samples. Phyllosphere microbiome composition varied across grazing intensity and healthy/diseased leaves (Figure 2). Among the epiphytic fungi, *Filobasidium* had the highest relative abundance in all treatments, followed by *Vishniacozyma*, *Zymoseptoria*, and *Sporobolomyces*. The relative abundance of *Parastagonospora*, and *Zymoseptoria* was higher in diseased leaves at each grazing intensity, while the opposite was true for *Sporobolomyces*. The relative abundance of *Rhodotorula* increased under heavy grazing. Among the epiphytic bacteria, the relative abundance of *Pantoea* was the highest in all treatments, where its relative abundance was as high as 90.99% in the G5H treatment, which was higher than all other treatments. The relative abundance of *Sphingomonas* was higher in all diseased leaves than in healthy leaves, and the relative abundance of *Enterococcus* was higher in G1 than in several other grazing intensities.

#### 2.2.2. Effects of Grazing on the Species Composition of Phyllospheric Micro-Organism

**Figure 3:**
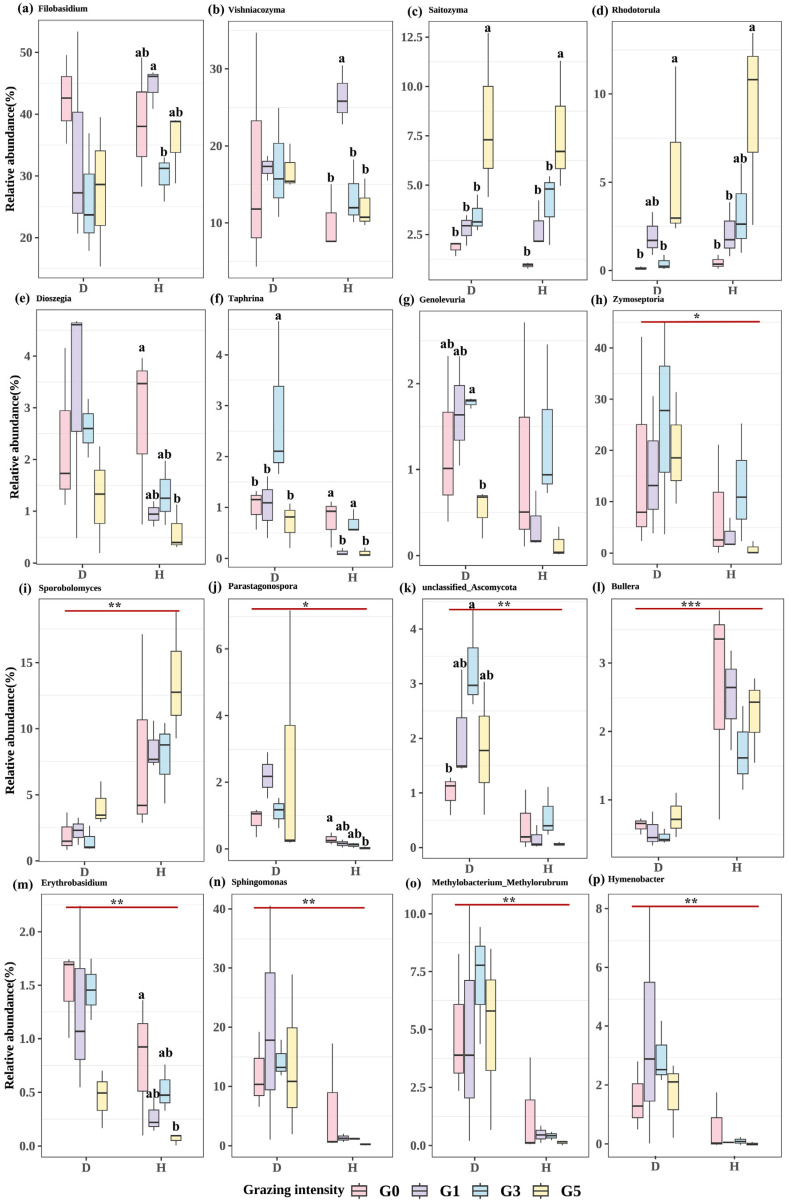
Box line plot of differences in relative abundance of epiphytic fungi (**a–m**) and epiphytic bacteria (**n–p**) selected micro-organisms. D represents leaf spot-diseased leaves and H represents healthy leaves. Different lowercase letters are used to indicate significant differences between different grazing intensities at *p* < 0.05, * indicates significant differences between leaf spot diseased leaves and healthy leaves, and the absence of labeled letters and * indicates no significant differences (* for *p* < 0.05, ** for *p* < 0.001, and *** for *p* < 0.0001). G0, 0 standard bovine units per hectare; G1, 0.23 standard bovine units per hectare; G3, 0.46 standard bovine units per hectare; and G5, 0.92 standard bovine units per hectare.

A one-way ANOVA was performed on the micro-organisms of the top 15 genera with the highest relative abundance at different grazing intensities (Figure 3). The results showed that among the epiphytic fungi, the relative abundance of *Rhodotorula*, *Saitozyma*, and *Sporobolomyces* increased gradually with increasing grazing intensity, but there was no significant difference between the gradients of *Sporobolomyces* (*p* > 0.05). There were no significant differences in the relative abundance of *Filobasidium*, *Vishniacozyma*, *Dioszegia*, *Parastagonospora*, and *Erythrobasidium* between different grazing intensities for diseased leaves (*p* > 0.05), but there were significant differences between grazing intensities of healthy leaves (*p* < 0.05). *Genolevuria* and unclassified *Ascomycota* were just the opposite, with significant differences between grazing intensities for diseased leaves (*p* < 0.05), but not for healthy leaves (*p* > 0.05), all showing a trend of now increasing and then decreasing as grazing intensity increased. The relative abundance of *Zymoseptoria* also tended to increase and then decrease between grazing intensities, but there was no significant difference (*p* > 0.05). The opposite was true for *Bullera*, which tended to decrease and then increase between grazing intensities. The relative abundance of *Taphrina* was significantly different between grazing intensities for both healthy and diseased leaves (*p* < 0.05). The relative abundance of diseased leaves was significantly higher at the G3 intensity than at the remaining grazing intensities (*p* < 0.05), and the relative abundance of healthy leaves was significantly higher at G0 and G3 than at G1 and G5 (*p* < 0.05). Among the epiphytic bacteria, there was no significant difference in the relative abundance of micro-organisms between different grazing intensities for both healthy and diseased leaves (*p* > 0.05), but the relative abundance of *Sphingomonas*, *Methylobacterium Methylorubrum*, and *Hymenobacter* showed a tendency to increase and then decrease with increasing grazing intensity (Figure 3).

#### 2.2.3. Impact of Leaf Spot Disease on Phyllosphere Microbiome Composition

A one-way ANOVA analysis of the top 15 genera of micro-organisms with the highest relative abundance in *L. chinensis* leaf spot diseased leaves versus healthy leaves. The results showed that among the epiphytic fungi, the relative abundance of *Zymoseptoria*, *Erythrobasidium*, *Parastagonospora*, and unclassified *Ascomycota* was significantly higher in diseased leaves than in healthy leaves (*p* < 0.05), while the relative abundance of *Bullera* and *Sporobolomyces* was significantly higher in healthy leaves than that of diseased leaves (*p* < 0.001). Among the epiphytic bacteria, the relative abundance of *Sphingomonas*, *Methylobacterium Methylorubrum*, and *Hymenobacter* was significantly higher in diseased leaves than in healthy leaves (*p* < 0.001) (Figure 3).

### 2.3. Environmental Factors and Phyllosphere Microbiome

**Figure 4:**
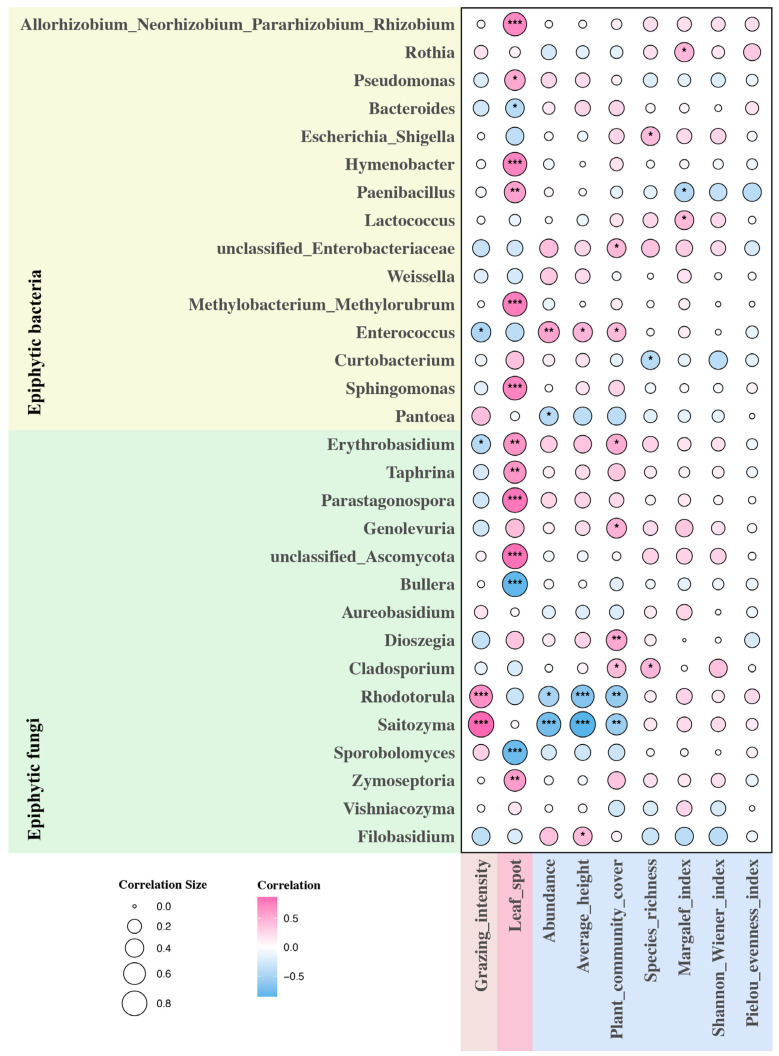
Heat map of correlation between environmental factors and epiphytic fungi and epiphytic bacteria. Red color is positive correlation, blue color is negative correlation, and the size of the circle represents the strength of the correlation. The darker the color and the larger the circle, the higher the correlation. * indicates a significant correlation (* is *p* < 0.05, ** is *p* < 0.01, *** is *p* < 0.001).

The top 15 micro-organisms in relative abundance at the level of epiphytic fungi and epiphytic bacterial genera were analyzed for correlation with environmental factors (Figure 4). Among the epiphytic fungi, the leaf spot of *L. chinensis* was significantly positively correlated with the relative abundance of *Zymoseptoria*, unclassified *Ascomycota*, *Parastagonospora*, *Taphrina*, and *Erythrobasidium* (*p* < 0.01), while it was significantly negatively correlated with the relative abundance of *Sporobolomyces* and *Bullera* (*p* < 0.001). Grazing intensity was significantly positively correlated with the relative abundance of *Saitozyma* and *Rhodotorula* (*p* < 0.001), while it was significantly negatively correlated with the relative abundance of *Erythrobasidium* (*p* < 0.05). Both the abundance of *L. chinensis* and the mean height of *L. chinensis* were significantly negatively correlated with the relative abundance of *Saitozyma* and *Rhodotorula* (*p* < 0.05), and the mean height of *L. chinensis* was also significantly positively correlated with the relative abundance of *Filobasidium* (*p* < 0.05). Plant community cover was also significantly negatively correlated with the relative abundance of *Saitozyma* and *Rhodotorula* (*p* < 0.01), and significantly positively correlated with the relative abundance of *Cladosporium*, *Dioszegia*, *Erythrobasidium*, and *Genolevuria* (*p* < 0.05). Species richness was significantly positively only correlated with the relative abundance of *Cladosporium* (*p* < 0.05) and did not have a significant effect on micro-organisms of any other genus (*p* > 0.05). The Margalef index and Shannon Wiener index were positively correlated with the relative abundance of most epiphytic fungi, but neither was significant (*p* > 0.05). Among epiphytic bacteria, *L. chinensis* leaf spot disease was associated with *Paenibacillus*, *Pseudomonas*, *Allorhizobium Neorhizobium Pararhizobium Rhizobium*, *Sphingomonas*, *Hymenobacter,* and *Methylobacterium Methylorubrum* were significantly positively correlated with the relative abundance (*p* < 0.05), but significantly negatively correlated with the relative abundance of *Bacteroides* (*p* < 0.05). Grazing intensity was negatively correlated with most of the epiphytic bacteria, but was only significantly negatively correlated with the relative abundance of *Enterococcus* (*p* < 0.05). Both *L. chinensis* abundance and mean height of *L. chinensis* were significantly positively correlated with the relative abundance of *Enterococcus* (*p* < 0.05), and in addition, *L. chinensis* abundance was significantly negatively correlated with the relative abundance of *Pantoea* (*p* < 0.05). Plant community cover was significantly and positively correlated with the relative abundance of *Enterococcus* and unclassified *Enterobacteriaceae* (*p* < 0.05). Species richness was significantly and negatively correlated with the relative abundance of *Curtobacterium* and significantly and positively correlated with the relative abundance of *Escherichia Shigella* (*p* < 0.05). The Margalef index was significantly negatively correlated with the relative abundance of *Paenibacillus* (*p* < 0.05), while it was significantly positively correlated with the relative abundance of *Rothia* and *Lactococcus* (*p* < 0.05). The Shannon Wiener index was not significantly correlated with any of these 15 epiphytic bacteria (*p* > 0.05).

### 2.4. Microbial Diversity

**Figure 5:**
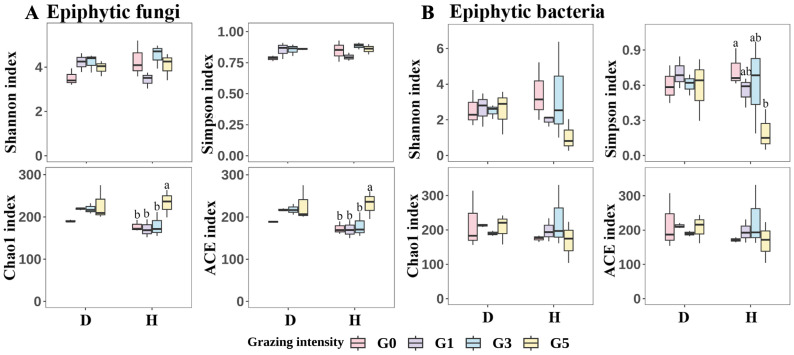
Box plots of differences in Alpha diversity indices for epiphytic fungi (**A**) and epiphytic bacteria (**B**). Different lowercase letters are used to indicate significant differences between grazing intensities (*p* < 0.05), and no labeled letters indicate no significant differences. D represents leaf spot-diseased leaves, and H represents healthy leaves. G0, 0 standard bovine units per hectare; G1, 0.23 standard bovine units per hectare; G3, 0.46 standard bovine units per hectare; G5, 0.92 standard bovine units per hectare.

**Figure 6:**
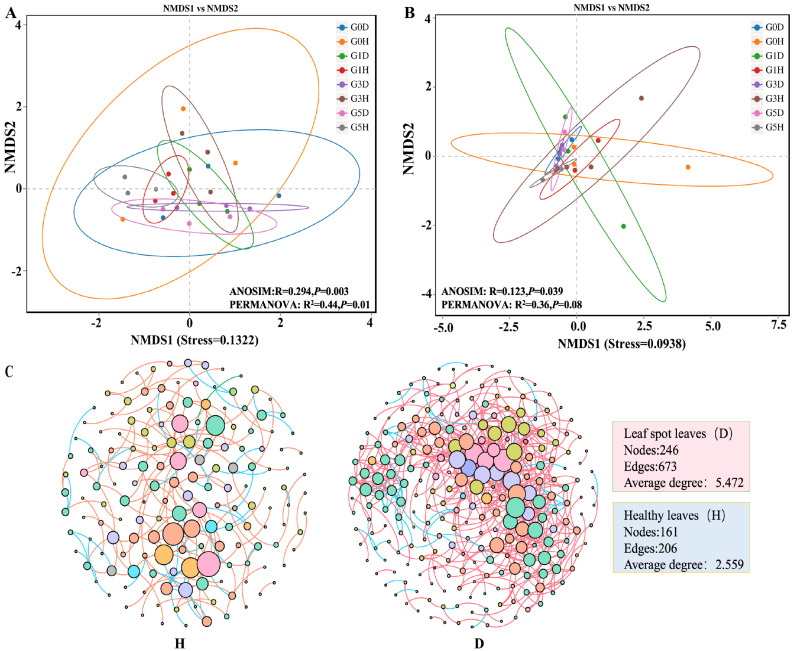
Nonmetric multidimensional scaling (NMDS) Diversity Analysis of Epiphytic Fungi (**A**) and Epiphytic Bacteria (**B**) Based on Bray-Curtis Distance. Each point in the figure represents a sample; different colors represent different subgroups; the oval circle indicates that it is a 95% confidence ellipse (i.e., if there are 100 samples in the sample group, 95 will fall in it). Phyllosphere microbiome co-occurrence network diagram of leaf spot diseased leaves and healthy leaves (**C**). In the figure, sphere nodes represent different OTUs, sphere size represents centrality scores, and sphere colors represent different gate levels to which the species belongs; lines represent correlations between the two species, and the color of the lines: orange represents positive correlations and blue represents negative correlations. D represents leaf spot-diseased leaves, and H represents healthy leaves. G0, 0 standard bovine units per hectare; G1, 0.23 standard bovine units per hectare; G3, 0.46 standard bovine units per hectare; and G5, 0.92 standard bovine units per hectare.

One-way analysis of variance (ANOVA) and Duncan’s test were performed to analyze the phyllosphere microbiome Alpha diversity of *L. chinensis* leaf spot leaves and healthy leaves at different grazing intensities, respectively, and a *t*-test was performed on the phyllosphere microbiome Alpha diversity of *L. chinensis* leaf spot leaves and healthy leaves (Figure 5). The results showed that among the epiphytic fungi, there was no difference in Shannon index and Simpson index between diseased and healthy leaves among different grazing intensities (*p* > 0.05), but the Shannon index and Simpson index of healthy leaves were slightly higher than those of diseased leaves under all grazing intensities except G1. There was also no significant difference in Chao1 index and ACE index between different grazing intensities for diseased leaves (*p* > 0.05), but both Chao1 index and ACE index were significantly higher for healthy leaves of G5 than for the remaining three grazing intensities (*p* < 0.05). In addition, Chao1 index and ACE index were higher in diseased leaves than in healthy leaves at all grazing intensities, except for G5H, where Chao1 index and ACE index were higher than in G5D, however, there was no significant difference in Chao1 index and ACE index between healthy and diseased leaves (*p* > 0.05). In epiphytic bacteria, there were no significant differences in Shannon index, Chao1 index and ACE index among all treatments (*p* > 0.05); Simpson index was significantly lower in healthy leaves in G5 than in G1 (*p* < 0.05). Nonmetric multidimensional scaling (NMDS) was used to analyze the β-diversity of phyllosphere microbiome community between treatments. The results showed some differences in phyllosphere microbiome community structure between treatments (Figure 6).

Based on the phyllosphere microbiome co-occurrence network diagram of leaf spot-diseased leaves versus healthy leaves, it was found that the connectivity and complexity of leaf spot-diseased leaves increased compared to healthy leaves (Figure 6C). Specifically, the nodes, the number of connections, and the number of connections per node were higher in the leaf spot-disease leaf network than in the healthy leaves.

### 2.5. Functional Gene Prediction

**Figure 7:**
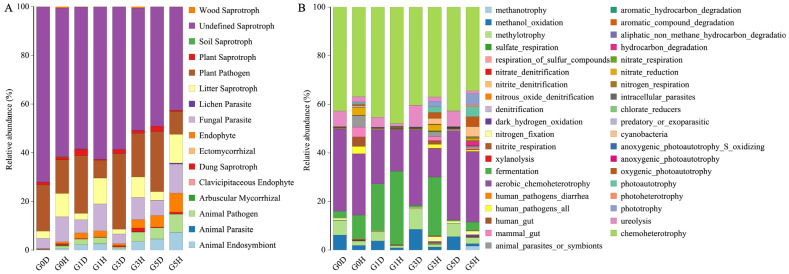
Stacked histograms of functional gene composition of epiphytic fungi (**A**) and epiphytic bacteria (**B**) under different treatments. G stands for grazing, the number represents the intensity of grazing, D stands for leaf spot diseased leaves, and H stands for healthy leaves. G0, 0 standard bovine units per hectare; G1, 0.23 standard bovine units per hectare; G3, 0.46 standard bovine units per hectare; and G5, 0.92 standard bovine units per hectare.

Funguild and FAPROTAX were used to predict functional genes for fungal and bacterial communities, respectively (Figure 7). Among epiphytic fungi, Undefined Saprotroph was the functional gene with the highest relative abundance among all treatments, with a decreasing trend with increasing grazing intensity, with G0D being the highest and G5H the lowest. Plant Pathogen and Fungal Parasite also accounted for a sizable proportion of the total, and the relative abundance of Plant Pathogen was lower in healthy leaves than in diseased leaves, with the highest relative abundance of G3D in diseased leaves and the lowest relative abundance of G3H in healthy leaves. Fungal Parasite was just the opposite, with higher relative abundance in healthy leaves than in diseased leaves; the relative abundance of Litter Saprotroph was also higher in healthy leaves than in diseased leaves. In addition to this Animal Endosymbiont, Animal Parasite, Animal Pathogen, Clavicipitaceous Endophyte, and Endophyte were all found to be in higher relative abundance in healthy leaves than in diseased leaves. The relative abundance of Plant Saprotroph was higher in diseased leaves than in healthy leaves, except for G0H, which was slightly higher than G0D. Among the epiphytic bacteria, chemoheterotrophy, and aerobic_chemoheterotrophy had the highest relative abundance. The relative abundance of aerobic chemoheterotrophy was higher in diseased leaves than in the healthy leaves, while chemoheterotrophy was higher in diseased leaves than in the healthy leaves, except for G1H, which was higher than G1D. The relative abundance of methanol oxidation, methylotrophy, and ureolysis was all higher in diseased leaves than in healthy leaves. Conversely, fermentation, nitrate denitrification, nitrite denitrification, nitrous oxide denitrification, denitrification, nitrite respiration, dark hydrogen oxidation, xylanolysis, human gut, mammal gut, hydrocarbon degradation, nitrate respiration, nitrate reduction, nitrogen respiration, predatory or exoparasitic, cyanobacteria, oxygenic photoautotrophy, photoautotrophy, and phototrophy were all in higher relative abundance in healthy leaves than in diseased leaves.

## 3. Discussion

Several studies have demonstrated that phyllosphere microbiomes are closely related to plant health, and grazing is one of the main utilization modes of natural grasslands. Studying the response of phyllosphere microbiome to grazing and diseases is important for the prevention and control of natural grassland diseases.

### 3.1. Effects of Grazing on Plant Communities and Leaf Spot Diseases

Many studies have highlighted the effects of grazing on natural grasslands, although the majority of attention has been on the functioning and diversity of grassland ecosystems [38,39]. It has been demonstrated in many studies that moderate grazing increases species richness and diversity in natural grasslands [40,41]. Our findings were consistent with these conclusions, as we found that grazing reduced the abundance and mean height of the dominant Hulunber plant, *Leymus chinensis*, and increased the species richness and diversity of the community as a whole. This might be due to the fact that cattle are fond of *L. chinensis*, and as grazing intensity increases, *L. chinensis* foraging rate increases, and the reduction of the dominant plant, *L. chinensis*, provides more space for the remaining nondominant plants to survive, such as sunlight, water, and carbon dioxide, and therefore promotes the biodiversity of the natural grassland [40,42]. It is commonly believed that high biodiversity mitigates the occurrence of plant diseases [43,44,45]. In addition, grazing significantly reduced the incidence and severity of leaf spot disease in *L. chinensis*. Thus, our results partly validated the idea that moderate grazing could be beneficial to the health of natural grasslands.

### 3.2. Effects of Leaf Spot Disease on Phyllosphere Microbiome

Our results showed that leaf spot disease strongly affected the composition of phyllosphere microbiome but exhibited no significant effect on microbial community diversity. Co-occurrence network analysis also showed more complex microbial activities in leaf spot-diseased leaves compared to healthy leaves, suggesting that phyllosphere microbiome in *L. chinensis* responds strongly to the disease when infested with pathogens. The result was in line with the findings of Li et al. (2022) [46]. In addition, the relative abundance of plant pathogens was significantly higher in diseased leaves than in healthy leaves, whereas it was significantly higher in healthy leaves than in the diseased leaves in the case of fungal parasites. It was implied that fungal parasites dominated the competition with plant pathogens in healthy leaves. The relative abundance of the epiphytic fungi *Zymoseptoria*, *Erythrobasidium*, *Parastagonospora*, and unclassified *Ascomycota* was significantly higher, while it was significantly lower in the diseased leaves of *Bullera* and *Sporobolomyces*. *Zymoseptoria* tritici and many species of *Parastagonospora* are the most destructive wheat pathogens [47,48]. The species of the genus *Zymoseptoria* have very strong host specialization, with some species infesting almost exclusively *Triticum* sp. and *Hordeum* sp. plants [49,50]. There are also species that can infest wild grasses, for example, *Zymoseptoria pseudotritici* can infest *Dactylis* sp., *Agropyron* sp., and *Phalaris* sp., while *Zymoseptoria ardabiliae* can infest *Lolium* sp., *Agropyron* sp., and *Elymus* sp. However, no species pathogenic to *Leymus* sp. have been identified to date [51]. In our results, the relative abundance of *Zymoseptoria* sp. in diseased leaves was much higher than that of *Parastagonospora* sp., which was the pathogen of *L. chinensis*. In addition, in healthy leaves, the relative abundance of *Zymoseptoria* sp. was significantly lower. We found that there were large tracts of farmland planted with wheat in the vicinity of our experimental site, and that *Zymoseptoria* sp. might be spread to the surface of *L. chinensis* by air currents.

The higher relative abundance of diseased leaves was possibly due to the disorganization of phyllosphere microbiome of *L. chinensis*, which facilitated the successful infestation by *Parastagonospora* sp. This created a favorable environment for microbial invasion, leading to a synergistic effect between the two pathogens, unlike the healthy leaves. *Bullera* and *Sporobolomyces* are two different yeasts that have the ability to compete for nutrients and produce lytic enzymes to protect plant health against phytopathogenic fungi, and are important biocontrol agents [52]. For example, *Bullera sinensis* FVF10, *Rhodotorula glutinis* had a good effect on *Rhizoctonia solani* [53]. Our findings, therefore, concluded that micro-organisms with increased relative abundance might have a crucial role in promoting the development of leaf spot disease in *L. chinensis*. In contrast, micro-organisms with decreased relative abundance, such as *Bullera* and *Sporobolomyces*, might be involved in inducing disease resistance in plants.

We also observed a significant increase in the relative abundance of the epiphytic bacteria *Sphingomonas*, *Methylobacterium Methylorubrum*, and *Hymenobacter* in leaves with leaf spot disease. Correlation analysis also showed that leaf spot disease was associated with the relative abundance of *Pseudomonas*, *Sphingomonas*, *Paenibacillus*, *Methylobacterium Methylorubrum*, *Allorhizobium Neorhizobium Pararhizobium Rhizobium*, and *Hymenobacter,* which were significantly positively correlated in relative abundance. Previous studies have demonstrated that plants recruit protective bacteria that enhance microbial activity to suppress pathogens, with *Pseudomonas*, *Paenibacillus*, and *Sphingomonas* being common genera of phytobiotic defenses [54,55,56]. *Pseudomonas* include multiple species and strains that inhibit plant pathogens and can promote plant growth, induce systemic resistance, degrade exogenous compounds, etc. [57,58]. For example, *Pseudomonas chlororaphis* subsp. *piscium* ZJU60 produces the antifungal compound PCN that inhibits fungal growth and virulence by inhibiting the HAT activity of the SAGA complex, which is antagonistic to eight plant pathogenic fungi [55]. In roots, *Paenibacillus polymyxa* emits the volatile organic compound C13, which was shown to induce systemic resistance [59], *Methylorubrum* promotes plant growth [60], and many species of *Hymenobacter* have radiation-resistant properties [61]. *Sphingomonas* showed significant potential to confer protection to the citrus phyllosphere against pathogen invasion through its iron-competition ability [46]. In addition, *Pantoea* was the dominant bacterial group in *L. chinensis*. Although not significant, the relative abundance was slightly higher in healthy leaves, and correlation analysis also showed that leaf spot disease was negatively correlated with its relative abundance. This could be because *Pantoea* can greatly inhibit the colonization of pathogenic bacteria, leading to a substantial reduction in pathogens [57,62].

Overall, an increase in the relative abundance of disease-causing micro-organisms leads to disease development, and an increase in the relative abundance of beneficial micro-organisms protects the plant. These two types of micro-organisms may regulate the occurrence of plant diseases through resource interactions, such as competition for nutrients. After disease development, plants would reduce the damage caused by pathogens by recruiting beneficial micro-organisms for themselves.

### 3.3. Effects of Grazing on Phyllosphere Microbiome

In our study, grazing intensity altered the relative abundance of micro-organisms, possibly by wounding plants through feeding, trampling, and other behaviors, creating conditions for microbial colonization and dispersal. Microbial dispersal and colonization can indirectly be affected by altering the structure of the plant community [11]. *Rhodotorula* and *Saitozyma* were significantly positively correlated with grazing, whereas they were significantly negatively correlated with *L. chinensis* abundance, mean height, and community cover, and positively correlated with species richness, the Shannon Wiener index, and Margalef index. It could be attributed to the fact that the decrease in *L. chinensis* abundance and mean height increased the distance between *L. chinensis*. Meanwhile, the decrease in community cover and the increase in plant diversity increased the distance between other plants and *L. chinensis* and further isolated *L. chinensis* populations through a diversity of plants, ultimately making microbial spread difficult [63,64]. Some micro-organisms, such as *Parastagonospora*, were negatively correlated with grazing and positively correlated with environmental factors, such as *L. chinensis* abundance. Probably because of the decrease in microbial abundance due to the removal of plant tissues by livestock feeding. Meanwhile, the increase in *L. chinensis* abundance, average height, and plant cover decreased the distance between *L. chinensis* populations, which was conducive to the dissemination of micro-organisms. The increase in diversity creates conditions for the spread of micro-organisms from other plants to *L. chinensis* [16,17,65].

## 4. Materials and Methods

### 4.1. Experimental Site

The study area was located 3 km east of the Xiertala cattle farm in Hailar Administrative District, Hulunber City, Inner Mongolia Autonomous Region. The region was known as the core of the *Leymus chinensis* meadow steppe in the transition zone from the western foothills of the Daxinganling Mountains to the Mongolian Plateau. The experiment relied on the long-term cattle grazing experimental platform of the National Hulunber Grassland Ecosystem Observation and Research Station (NHGEORS) (49°32′–49°34′ N, 119°94′–119°96′ E), at an altitude of 670–677 m. The climate was temperate and semiarid continental, with an average annual precipitation of 350–400 mm, and the soil type was mainly calcareous black soil or calcareous chestnut soil. The vegetation type was meadow steppe, and the main established species was *L. chinensis*. The main crops in the Xiertala, where the research station was located, include wheat, barley, corn, oilseed rape, alfalfa, oats, and sugar beets, with a total area of approximately 21,300 ha, of which grain crops accounted for approximately 30%.

### 4.2. Experimental Plot Design

The grazing experiment in this study area started in 2009, and the livestock were Sanhe cattle. The trial area was designed as a randomized block design with six grazing intensities, three replications, and a total of 18 plots comprising a total area of 90 ha, each with an area of 5 ha (300 m × 167 m). Grazing intensities were 0, 0.23, 0.34, 0.46, 0.69, and 0.92 standard bovine units per hectare (AU/ha). Each grazing intensity level corresponded to a specific number of Sanhe cattle (250–300 kg calves) per plot, i.e., 0, 2, 3, 4, 6, and 8. In total, there were 69 Sanhe cattle. The grazing period was from 1 June to 1 October each year, and the livestock were in the grazing plots day and night (24 h) throughout the grazing period. The grazing period lasted 120 days, during which drinking water was hand-supplied at regular intervals. In this experiment, disease survey and sampling were conducted on plots with grazing intensities of 0, 0.23, 0.46, and 0.92 standard bovine units per hectare (AU/ha), denoted as G0, G1, G3, and G5.

### 4.3. Plant and Disease Incidence Investigation

Morphological and molecular biological identification were used to identify the pathogen, and the dominant pathogen causing leaf spot disease of *L. chinensis* in this region was *Parastagonospora nodorum*, consistent with the findings of Yawen Zhang [5]. The field survey and sampling were conducted in August 2023, during the peak growth period of plants. Each time, eight 0.5 m × 0.5 m sample squares were selected along the diagonal of each plot (avoiding the edge of the plot), for a total of 8 × 12 = 96 sample squares, to investigate the cover, height, abundance, and frequency of all plants in the sample squares. The number of cases and severity of leaf spot disease in each sample square (the grading standard for leaf spot disease was adopted from the grading method of Nan, (1998) [66]) were recorded, and the incidence rate and severity of leaf spot disease in *L. chinensis* were calculated. Based on the vegetation survey data of the samples, the species importance value (V), Margalef index (MI), and Shannon-Wiener index (SWI) were calculated.

Leaf spot disease grading scale: Grade 0: asymptomatic; Grade 1: spot area < 5% of leaf area; Grade 2: spot area accounted for 6–20% of leaf area; Grade 3: spot area accounted for 21–40% of leaf area; Grade 4: 41–70% of leaf blade area covered by lesions; Grade 5: spot area > 71% of leaf area.

(1) $$\mathrm{Disease}\;\mathrm{severity}=\frac{(\sum \mathrm{grade}\times \mathrm{number})\times 100}{\mathrm{total}\;\mathrm{number}\times \mathrm{top}\;\mathrm{grade}}\;V=\frac{Pi+Cr+Hr+Dr}{4}\;MI=\frac{SR-1}{\ln N}\;SWI=-\sum V\ln V$$

where Pi = Ni/N, Hr is the relative height, Cr is the relative cover, Dr is the relative frequency, Ni is the number of individuals of species i, N is the total number of individuals in the community, and SR is the number of species in the community, i.e., species richness.

### 4.4. Phyllospheric Micro-Organism Sampling and Processing

After the field survey, a total of 20 leaves from both disease-affected (D) and nonaffected (H) *L. chinensis* in each plot were randomly selected in the evening when it was almost dark. The samples were put into a low-temperature preservation box and immediately transported to the laboratory [46]. The leaves were then cut with sterilized scissors and put into 50-mL sterile centrifugal tubes.

Collection of plant epiphytic micro-organisms: Plant leaves were weighed as 5 g on sterile filter paper on an ultra-clean bench and transferred with sterile forceps to a sterile centrifuge tube filled with sterile PBS buffer. The centrifuge tube was subjected to ultrasonic oscillation (1 h, 200 rpm, 20 °C) for 1 min and vortex for 30 s, alternately, and repeated five times. The epiphytic micro-organisms on the leaf surface were washed into the buffer. The buffer was transferred to a sterilized glass funnel, and the washing solution was filtered by a 0.22 μm sterile membrane filter to obtain the filter membrane enriched with micro-organisms. The membrane was then cut into 2 × 2 mm pieces with sterilized scissors and stored in a sterile freezing tube at −80 °C.

**Figure 8:**
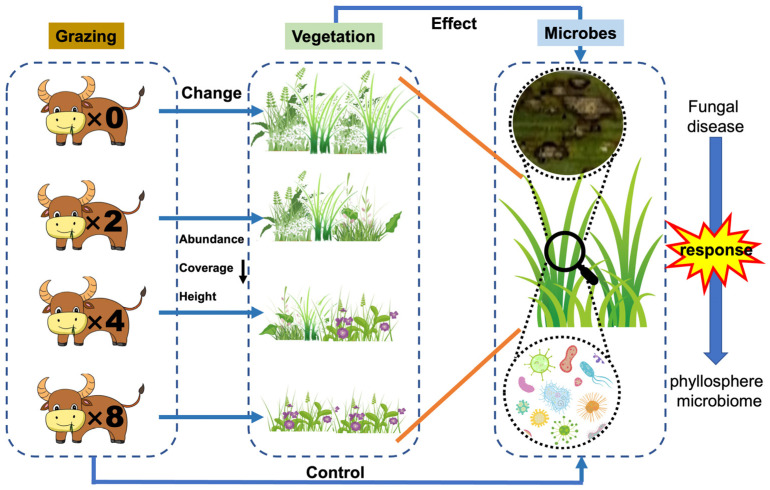
Grazing-disease—phyllosphere microbiome schema (the number indicates the number of cattle in each plot).

DNA extraction and sequencing: Total genomic DNA was extracted from filter membranes and leaf samples using the TGuide S96 Magnetic Soil/Stool DNA Kit (Tiangen Biotech (Beijing) Co., Ltd., Beijing, China), following the manufacturer’s instructions. The quality of extracted DNA was detected by 1.8% agarose gel electrophoresis, and the concentration and purity of DNA were detected by NanoDrop 2000 UV-vis spectrophotometer (Thermo Scientific, Wilmington, NC, USA). Then, the extracted DNA was subjected to PCR amplification of the v3 + v4 region of bacterial 16SrRNA and the fungal ITS1 region. The fungal primers were ITS1F (5′-CTTGGTCATTTAGAGGAAGTAA-3′) and ITS2 (5′-GCTGCGTTCTTCATCGATGC-3′), and the epiphytic bacterial primers were 338F (5′-ACTCCTACGGAGGCAGCA-3′) and 806R (5′-GGACTACHVGGGTWTCTAAT-3′) [67]. Fungal PCR reaction conditions were: predenaturation at 95 °C for 5 min; denaturation at 95 °C for 1 min, annealing at 55 °C for 30 s, elongation at 72 °C for 1 min, 8 cycles; denaturation at 95 °C for 1 min, annealing at 60 °C for 30 s, elongation at 72 °C for 40 s, 20 cycles; and finally elongation at 72 °C for 7 min. The PCR reaction conditions for epiphytic bacteria were: predenaturation at 95 °C for 5 min; denaturation at 95 °C for 30 s, annealing at 50 °C for 30 s, elongation at 72 °C for 40 s for 20 cycles; and finally elongation at 72 °C for 7 min. The amplification products were purified using the Omega DNA purification kit (Omega Inc., Norcross, GA, USA) and quantified using Qsep-400 (BiOptic, Inc., New Taipei City, China) for quantification. Amplicon libraries were bipartite sequenced (2 × 250) on an Illumina novaseq6000 (Beijing Biomarker Technologies Co., Ltd., Beijing, China). The raw data obtained from sequencing was trimmed using Trimmomatic (version 0.33) and Cutadapt (version 1.9.1). USEARCH (version 10) was used to perform double-ended sequence splicing on the quality-controlled data, and then chimeras were removed to obtain high-quality sequences using UCHIME (version 8.1). Sequences were clustered into OTUs by USEARCH (v 10) based on a 97% similarity threshold. Taxonomic annotation of bacterial and fungal OTUs based on QIIME2 using Silva (v. 138) and UNITE (v. 7.2) as reference databases, as well as calculation of microbial abundance and Alpha diversity index. Microbial community differences were analyzed based on Bray-Curtis distance coefficients, and fungal functional diversity was predicted using Funguild for functional annotation. Bacteria were predicted using FAPROTAX for functional annotation, and all data were analyzed and plotted using SPSS. 27 with R4.3.2. The research idea is shown in Figure 8.

## 5. Conclusions

In this study, it was demonstrated that grazing significantly reduced the occurrence of *Leymus chinensis* leaf spot disease and affected plant communities. The phyllosphere microbiome responds strongly to the occurrence of *L. chinensis* leaf spot disease and grazing. The results showed that grazing increased the relative abundance of beneficial micro-organisms and decreased the relative abundance of disease-causing micro-organisms on the surface of *L. chinensis* leaves. The relative abundance of some antagonistic bacteria against pathogens increased after the development of leaf spot disease. In addition, the relative abundance of micro-organisms, such as *Bullera* and *Sporobolomyces*, was significantly higher on the surface of healthy leaves than that on the surface of diseased leaves, and these micro-organisms could be relevantly studied as potential biocontrol fungal agents. By studying the response of the phyllosphere microbiome to both grazing and leaf spot disease, it could be concluded that grazing is involved in maintaining the health of *L. chinensis* by modulating microbial community composition and structure, either directly or indirectly. In the grassland ecology of the region, the optimum grazing intensity for controlling leaf spot disease of *L. chinensis* was 0.92 standard bovine units per hectare (AU/ha). However, in production practice, it needs to be analyzed in combination with grass productivity, edible forage production, and other comprehensive analyses.

## Data Availability

The datasets used and/or analyzed during the current study are available from the first author on reasonable request. The sequencing data have been submitted to the NCBI Sequencing Read Archive (SRA) under the bio-project PRJNA1114935.
